# Framing susceptibility in a risky choice game is altered by galvanic vestibular stimulation

**DOI:** 10.1038/s41598-017-02909-4

**Published:** 2017-06-07

**Authors:** Nora Preuss, Roger Kalla, Rene Müri, Fred W. Mast

**Affiliations:** 1 Department of Psychology, University of Bern, 3012 Bern, Switzerland; 2 Center for Cognition Learning and Memory, University of Bern, 3012 Bern, Switzerland; 3 Department of Neuroscience, Karolinska Institutet, 17177 Stockholm, Sweden; 4 Division of Cognitive and Restorative Neurology, Department of Neurology, Inselspital, University Hospital Bern and University of Bern, 3010 Bern, Switzerland; 5 Department of Neurology and German Center for Vertigo and Balance Disorders, Ludwig-Maximilians-University Munich, Marchioninistr. 15, 81377 Munich, Germany; 6 Gerontechnology and Rehabilitation Group, University of Bern, 3008 Bern, Switzerland; 7 Perception and Eye Movement Laboratory, Department of Neurology and Clinical Research, Inselspital, University Hospital Bern and University of Bern, 3010 Bern, Switzerland

## Abstract

Recent research provides evidence that galvanic vestibular stimulation (GVS) has a modulating effect on somatosensory perception and spatial cognition. However, other vestibular stimulation techniques have induced changes in affective control and decision making. The aim of this study was to investigate the effect of GVS on framing susceptibility in a risky-choice game. The participants were to decide between a safe and a risky option. The safe option was framed either positively or negatively. During the task, the participants were exposed to either left anodal/right cathodal GVS, right anodal/left cathodal GVS, or sham stimulation (control condition). While left anodal/right cathodal GVS activated more right-hemispheric vestibular brain areas, right anodal/left cathodal GVS resulted in more bilateral activation. We observed increased framing susceptibility during left anodal/right cathodal GVS, but no change in framing susceptibility during right anodal/left cathodal GVS. We propose that GVS results in increased reliance on the affect heuristic by means of activation of cortical and subcortical vestibular-emotional brain structures and that this effect is modulated by the lateralization of the vestibular cortex.

## Introduction

It has long been proposed that vestibular processing, cognition, and emotion are linked (for overview see ref. 1). Clinical studies have reported high comorbidities between vestibular and emotional disorders^2, 3^ and recent experimental studies have demonstrated the influence of vestibular information on emotion processing and vice versa^4–9^. First experimental evidence was provided by means of caloric vestibular stimulation (CVS). CVS is a common technique in clinical vestibular diagnostics and it involves irrigating the outer ear canal with hot or cold water/air. Thermal stimulation of the outer ear canal predominantly affects the endolymphatic fluid in the horizontal semicircular canal, which in turn modulates the activation of the vestibular nerve. In two recent studies, we were able to show that CVS modulates affective control and decision making in healthy participants^7, 8^. We proposed that this effect is due to activation of vestibular cortical areas, which functionally overlap with areas involved in emotion processing. Galvanic vestibular stimulation (GVS) is another way to stimulate vestibular cortical areas. GVS involves placing an electrode of one polarity over the left mastoid and an electrode of another polarity over the right mastoid (i.e., behind the participant’s ears; for an overview see ref. 10). The applied currents are weak, usually between 0.8 and 2.0 mA. In contrast to CVS, GVS modulates the activity of the entire vestibular nerve, including afferents from the semicircular canals and the otoliths^10, 11^. Left anodal/right cathodal GVS is comparable to left cold or right warm CVS as it induces an imbalance of activation in favor of the right vestibular nerve. Right anodal/left cathodal GVS leads to less lateralized activation and is comparable to right cold or left warm CVS. Coactivation of the vestibular end organs results in activation of a widespread vestibular network including insular cortex, temporoparietal cortex, anterior cingulate cortex, basal ganglia, superior temporal gyrus, and temporoparietal junction^12–14^. Interestingly, left anodal/ right cathodal ear GVS induces more right-hemispheric vestibular cortical activations whereas the reverse polarity induces bilateral activations^10, 14^. Dieterich *et al*.^15^ were able to show a dominance of vestibular cortical functioning in the non-dominant hemisphere. In right-handed participants, cortical and subcortical activations were predominant in the right hemisphere, whereas a reverse pattern was found in left-handed participants. Dieterich *et al*.^15^ concluded that the vestibular system and its hemispheric dominance may influence handedness (see also ref. 16).

Despite the fact that hemispheric asymmetries have been a controversial subject to many researchers^17–19^, several studies suggest that the right hemisphere is more involved in the processing of global or contextual information while the left hemisphere is more involved in the local processing of details^19, 20^. In addition, the left hemisphere is mainly associated with language processing while the right hemisphere is mainly associated with the processing of spatial information^21^. Indeed, hemispheric differences have not only been proposed to play a role in cognitive functions but also in emotion processing^22–24^, with the right hemisphere being specialized for recognizing, controlling, and expressing emotions. Moreover, the right hemisphere is thought to be involved in the processing of negative emotions (“withdrawal response”) while the left hemisphere is thought to be involved in the processing of positive information (“approach response”)^25^.

Researchers have also proposed that there are hemispheric differences in risky-choice framing^26^. Framing refers to the wording of a task and has been found to influence a person’s response. Thus, a person’s response differs when a given task is presented in a positive frame (as a gain) as opposed to a negative frame (as a loss). The original risky-choice framing paradigm can be traced back to Tversky and Kahneman^27^ and is known as the “Asian disease problem”: “Imagine that the U.S. is preparing for the outbreak of an unusual Asian disease, which is expected to kill 600 people. Two alternative programs to combat the disease have been proposed. Assume that the exact scientific estimate of the consequence of the programs are as follows: …”^27^, p. 453. When asked to choose between the following two options, people tend to select the first (risk averse) over the second (risk seeking) option: “If Program A is adopted, 200 people will be saved. If Program B is adopted, there is a 1/3 probability that 600 people will be saved and a 2/3 probability that no people will be saved. Which of the two programs do you favor”? In contrast, if the options are framed negatively, people tend to select the second (risk seeking) over the first (risk averse) option: “If Program A is adopted, 400 people will die. If Program B is adopted, there is a 1/3 probability that nobody will die and a 2/3 probability that 600 will die. Which of the two programs do you favor”?^27^, p. 453. The expected outcome is identical in both alternatives; the only difference is the wording (“people will be saved” or “people will die”). Nevertheless, the wording matters and modulates people’s choice: People tend to make risk-averse choices when the options are presented in a positive frame (“people will be saved”) and risk-seeking choices when the options are presented in a negative frame (“people will die”). Interestingly, using a motor task, McElroy and Seta^26^ showed that the manifestation of the framing effect depended on which hemisphere was activated: They observed a framing effect when they selectively activated the right hemisphere by left finger tapping, but not when they selectively activated the left hemisphere by right finger tapping. They concluded that, when the participants’ right hemisphere was activated, they focused more on the context to solve the task, which made them more susceptible to the frame. In contrast, when participants’ left hemisphere was activated, they used a more analytical approach and relied less on contextual information to solve the task, which made them less susceptible to the frame. It is, however, not clear whether left finger tapping induced or strengthened framing susceptibility as a no-finger tapping control condition was missing. In another study, Gallagher and Dagenbach^17^ embedded the Asian disease problem in either a low-frequency or a high-frequency sound. According to the double-filtering-by-frequency hypothesis^21^, lower-frequency information is processed more efficiently in the right hemisphere and higher-frequency information is processed more efficiently in the left hemisphere^17^ for review. The framing effect only manifested in the lower-frequency condition, again suggesting an involvement of the right hemisphere. Moreover, Jasper *et al*.^28^ found differing levels of framing susceptibility in consistent and inconsistent handers: Inconsistent handers were more responsive to framing information. Inconsistent handedness is associated with greater functional access to the right hemisphere whereas a pronounced right handedness results in less interhemispheric interaction^28^.

The aim of the present study was to examine framing susceptibility during GVS. As summarized above, GVS can be used as a non-invasive brain stimulation technique to activate several cortical and subcortical regions that are involved in vestibular and emotion processing as well as decision making. Furthermore, lateralization of the activation pattern differs as a function of the polarization of the electrodes. In the present experiment, the participants performed a computerized version of a risky-choice framing task adapted from^29^ while they were exposed to left anodal/right cathodal GVS or sham stimulation (Experiment 1) and right anodal/left cathodal GVS or sham stimulation (Experiment 2). Left anodal/right cathodal GVS activates right-hemispheric vestibular cortex more^14^, so we expected to observe an increase in participants’ framing susceptibility following left anodal/right cathodal GVS compared to sham stimulation. Right anodal/left cathodal GVS results in a less lateralized brain activation pattern^14^, so we did not expect to observe a change in participants’ framing susceptibility following right anodal/left cathodal GVS compared to sham stimulation. The results will extend the existing evidence for the involvement of the vestibular network in a wide range of cognitive tasks by showing that GVS also modulates risky decision making.

## Results

### Experiment 1

**Figure 1:**
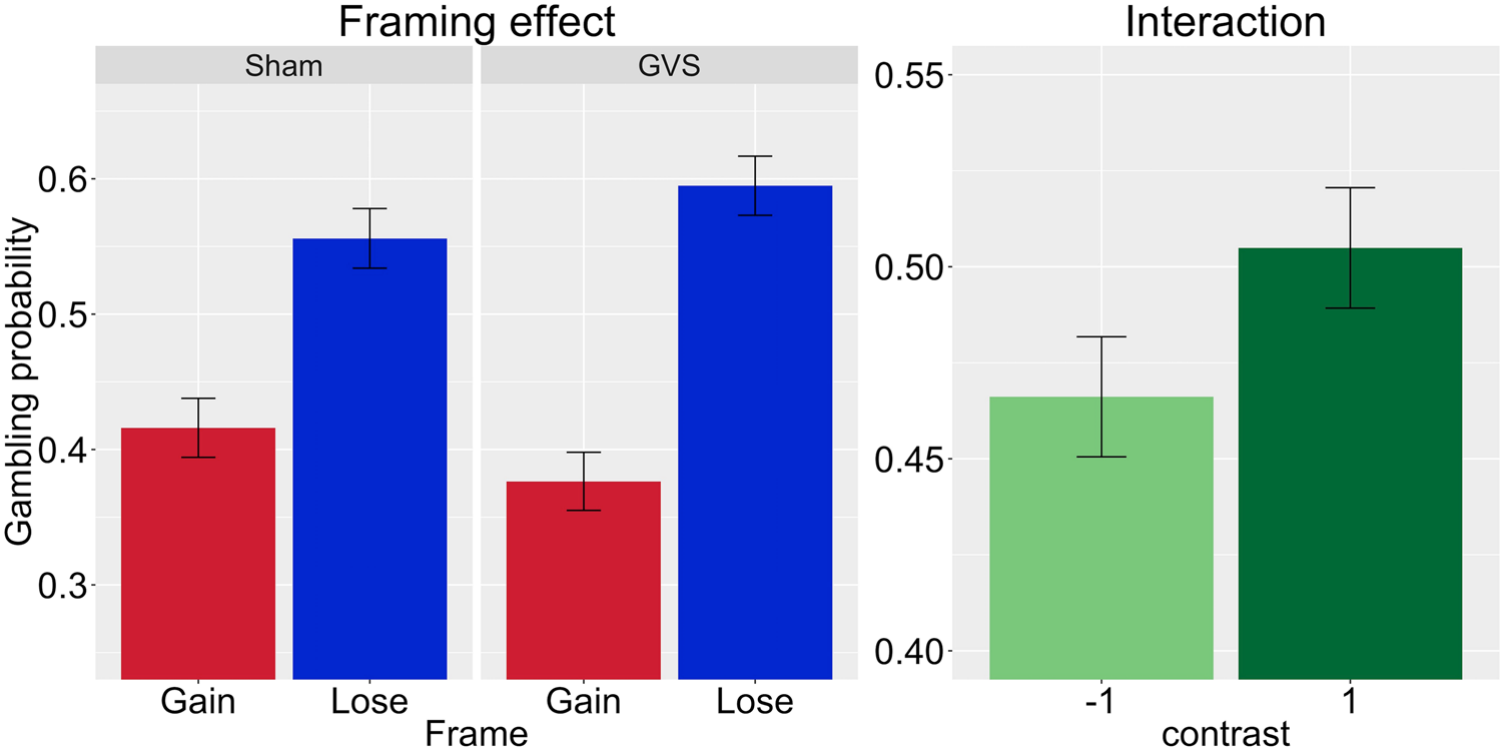
Results of Experiment 1: The left figure shows the framing effect as a function of galvanic vestibular stimulation (left anodal/right cathodal GVS) condition. The right figure shows the interaction term using contrast coding. Using a hierarchical logistical regression model, we found a significant interaction between stimulation and framing condition in Experiment 1.

The analysis revealed a 41.3% probability of gambling in the reference category (sham stimulation, positive frame), γ_00_ = −0.351, SE = 0.14, z = −2.54, p = 0.01. As expected, when the frame was negative (in the sham stimulation condition), the participants’ gambling probability increased significantly to 56.1%, γ_20_ = 0.6, SE = 0.15, z = 4.1, p < 0.001. Stimulation condition had no significant influence on gambling probability, γ_10_ = −0.24, SE = 0.22, z = −1.07, p = 0.28. Importantly, the interaction between stimulation condition and frame was significant, γ_30_ = 0.414, SE = 0.19, z = 2.17, p = 0.015 (one-sided, p = 0.03 if tested two-sided). Thus, framing susceptibility increased during left anodal/right cathodal GVS. The results are illustrated in Fig. 1. See full description of the hierarchical model in the supplementary material.

### Questionnaires

In Experiment 1, both the sham and the GVS condition were rated as being equally pleasant, T(15) = 1.77, p = 0.1. Furthermore, there were no differences between GVS and sham stimulation in the negative and positive affect ratings that were measured after stimulation: positive mood T(15) = 1.69, p = 0.11; negative mood T(15) = −1.06, p = 0.31.

### Experiment 2

**Figure 2:**
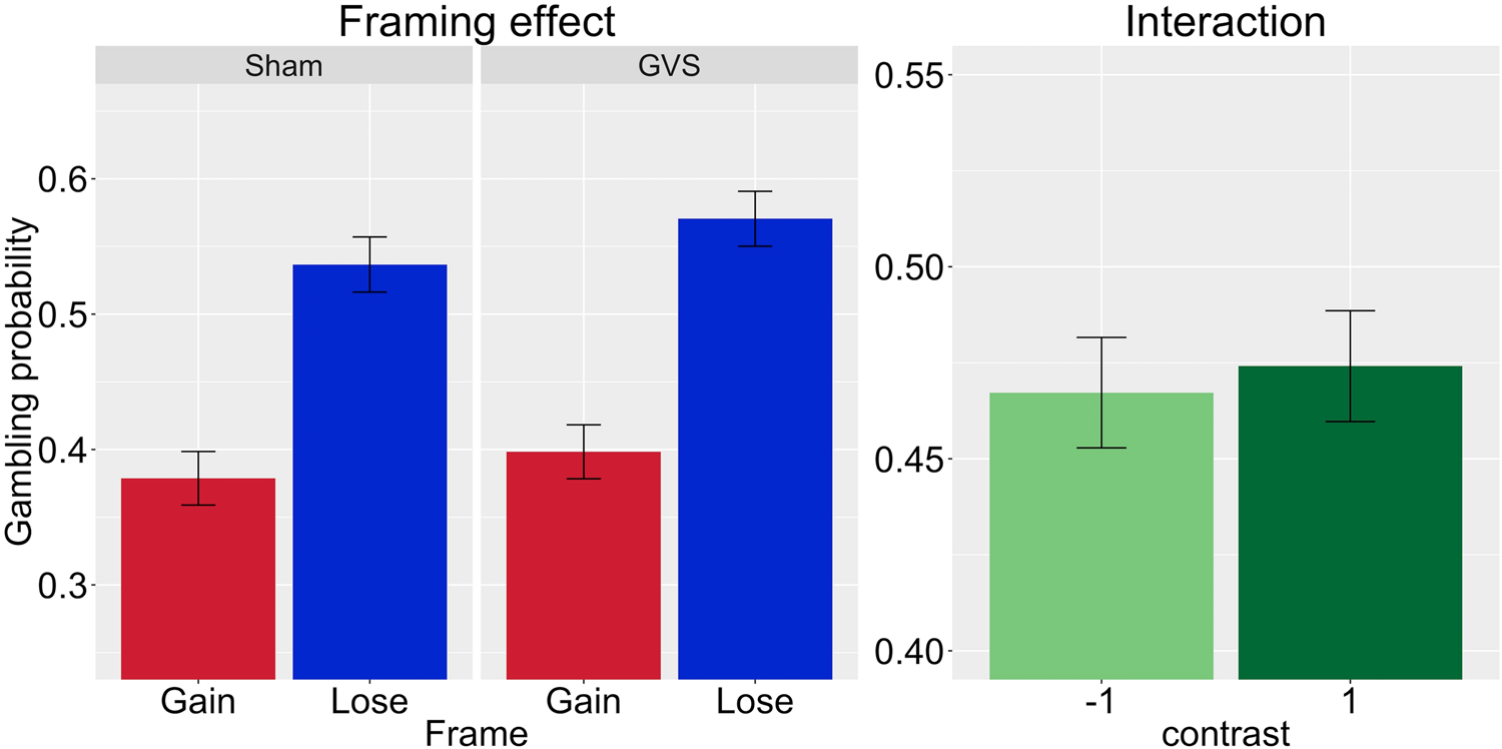
Results of Experiment 2: The left figure shows the framing effect as a function of galvanic vestibular stimulation (right anodal/left cathodal GVS) condition. The right figure shows the interaction term using contrast coding. Using a hierarchical logistical regression model, we found no significant interaction between stimulation and framing condition in Experiment 2.

The analysis revealed a 37.1% probability of gambling in the reference category, γ_00_ = −0.526, SE = 0.17, z = −3.09, p < 0.001. As expected, the participants’ gambling probability increased significantly to 54.3%, γ_20_ = 0.7, SE = 0.13, z = 5.5, p < 0.001 when the frame turned negative (in the sham stimulation condition). As in Experiment 1, stimulation condition had no significant influence on gambling probability, γ_10_ = 0.08, SE = 0.18, z = 0.44, p = 0.66, and, importantly, stimulation condition and frame did not interact, γ_30_ = 0.05, SE = 0.17, z = 0.3, p = 0.77. Right anodal/left cathodal GVS did not influence framing susceptibility. The comparison of the threshold values from Experiment 1 and 2 revealed no significant difference between the two experiments, t = −1.14, df = 20.11, p = 0.27. Our results are illustrated in Fig. 2. For a full description of the hierarchical model, see the supplementary material.

### Questionnaires

In Experiment 2, sham and GVS condition were rated as being equally pleasant, T(18) = 0.18, p = 0.86. Furthermore, there were no differences between GVS and sham stimulation in the negative and positive affect ratings that were measured after stimulation: positive mood T(18) = −0.59, p = 0.56; negative mood T(18) = 1.71, p = 0.11.

## Summary

The results show that participants’ gambling probability highly depended on how the information presented to them was framed. As expected, the participants preferred the safe option in the positive framing condition, but preferred the gambling option when the information was presented in a loss frame. Interestingly, left anodal/right cathodal GVS (Experiment 1) had an enhancing effect on the framing susceptibility whereas right anodal/left cathodal GVS exerted no influence on framing susceptibility. This finding is in accordance with our hypothesis that left anodal/right cathodal GVS would result in more pronounced right-hemispheric activation of the vestibular network and that right anodal/left cathodal GVS would result in less lateralized activation of vestibular projection areas.

## General Discussion

The aim of the present research was to examine the influence of GVS on framing susceptibility. We were able to show that left anodal/right cathodal but not right anodal/left cathodal GVS exerted a modulating effect on framing susceptibility. When the participants were exposed to left anodal/right cathodal GVS, their probability of gambling increased more in loss trials and decreased more in gain trials as compared to the sham stimulation condition. We attribute this finding to the lateralized activation pattern induced by GVS. Left anodal/right cathodal GVS activates more right-hemispheric areas of the vestibular network^14^, which includes the insular cortex, anterior cingulate cortex, and temporoparietal junction^12–14^. In contrast, right anodal/left cathodal GVS results in a more bilateral activation pattern^14^. The present results are in line with previous findings showing increased framing susceptibility when the right hemisphere is activated^17, 26, 28^.

De Martino *et al*.^29^ proposed that the framing effect is a behavioral bias that arises from an affect heuristic guided by the emotional system^30^. Based on our results, it is likely that left anodal/right cathodal GVS activates the emotional system and promotes an affect heuristic, resulting in a stronger susceptibility to emotional information. Neuroimaging studies provide evidence that the framing effect is modulated by structures such as the orbitofrontal and medial prefrontal cortex, the anterior cingulate cortex, and the amygdala^29^. However, it is noteworthy that the modulating effect of GVS on framing susceptibility cannot be solely attributed to a general activation of right-hemispheric structures. Indeed, De Martino, *et al*.^29^ reported that enhanced activity of the right orbitofrontal cortex and the ventromedial prefrontal cortex is correlated with decreased susceptibility to the framing effect. The amygdala, in contrast, plays a central role in mediating the framing effect. Amygdala activity is high when the participant chooses the safe option in the gain frame and the gamble option in the loss frame. Amygdala activity is significantly lower when the participant chooses an option that conflicts with the affect heuristic. Carmona, *et al*.^31^ pointed out the crucial role of the anterior cingulate cortex (ACC), being a key structure in emotion processing that is also activated by GVS. ACC constitutes the bridge, or the interface, that connects the prefrontal cognitive division with the more subcortical emotional division^31^. It monitors the conflict between the two systems. It is thus possible that GVS creates an imbalance between emotional and cognitive processes by activating more posterior vestibular-emotional areas such as the insular and anterior cingulate cortex. Carmona *et al*.^31^ pointed out that frontal regions exert control over posterior systems and that creating an imbalance within the system influences vestibular as well cognitive processes depending on the extent of frontal regulatory functions. We propose that creating an imbalance in favor of the posterior, more emotional system results in a greater reliance on the affect heuristic and therefore increases framing susceptibility. Based on the lateralization of the vestibular cortex to the non-dominant hemisphere, this effect is only evident in left-anodal/right-cathodal GVS. Further imaging studies will be required to confirm this assumption.

In this study, we were able to demonstrate the influence of GVS on emotional processes and risky decision making. Previous studies had shown that vestibular stimulation by means of CVS has an effect on affective control and mood^7^, decision making^8^, unrealistic optimism^9^, and on manic symptom severity^4^. So far, experimental studies using GVS to investigate cognitive and specifically emotional processes are still scarce. Until now, researchers have mainly examined the influence of GVS on bodily awareness^4, 5^, mental transformation abilities^32^, (hemi-)spatial attention^33^ and visual memory recall^34, 35^. Given the widespread cortical and subcortical activations of the vestibular system, it is not surprising that it is intertwined with emotional processes. The results of this study provide first evidence that GVS may serve as an effective tool to better investigate the overlap between cognitive and affective neuroscience.

A shortcoming of the present study is lacking information about whether participants experienced any difference between the experimental and sham condition. However, the stimulation was relatively weak and none of the participants made any remarks regarding differences after finishing the experimental procedure. Furthermore, only right-handed participants were included in the study. Previous studies, almost exclusively investigated vestibular influences on higher order cognitive processes in right-handed participants. Interestingly, the lateralization of vestibular cortical functions to the non-dominant hemisphere has been proposed to influence handedness^15, 16^. This rises the questions whether GVS is also an effective technique in modulating cognitive processes in left-handed participants and whether this effect is comparable or inverted. Future studies should therefore also investigate the modulating effect of GVS on higher order cognitive processes in left-handed participants.

The advantage of GVS over other non-invasive brain stimulation techniques – such as transcranial direct current stimulation (tDCS) – is that GVS also modulates subcortical emotional-vestibular structures (e.g., insula or ACC). Thus, even though GVS induces an activation of the afferent vestibular nerve, a possible spreading to other networks cannot be excluded. Future studies in combination with neuroimaging techniques are needed to better investigate the neuronal mechanisms that underlie the cognitive and affective processes that are commonly modulated by GVS.

## Material and Methods

### Participants

We tested a total of 44 participants, 22 in Experiment 1 (age = 23.1, SD = 2.17, male = 7) and 22 different participants in Experiment 2 (age = 22.28 ± 3.18, male = 4). All participants were right-handed (Edinburgh Handedness Questionnaire)^36^ and gave informed consent prior to the experiment. The experiment was conducted in accordance with the local ethics guidelines and the experimental procedure was approved by the local ethics committee (University of Bern).

### Galvanic vestibular stimulation

In Experiment 1, we placed the anode over the participants’ left mastoid and the cathode over their right mastoid (i.e., behind their ears). GVS was applied using a DC-Stimulator (neuroConn GmbH, Ilmenau, Germany). The participants differed in their sensitivity to galvanic vestibular stimulation. Therefore, an individual threshold detection task was performed prior to the actual experiment. A current, increasing in 0.25-mA steps from 0–2 mA (duration 60 s), was applied and participants were to report when they started to feel dizzy. The procedure was repeated twice and the vestibular threshold was determined by the lower value of the two minus 0.25 mA in order to ensure that the participants would be unaware of whether they were in the real or sham stimulation condition. The mean vestibular threshold was 1.009 mA (SD = ±0.338).

Each experimental stimulation block lasted for 15 minutes and participants were exposed to two stimulation blocks in total: one GVS and one sham GVS block. Half of the participants started with real GVS, and half of the participants started with sham GVS. During sham GVS, we increased the current for a duration of 30 s until the individual threshold was reached. Then the current remained stable for 15 s and decreased back to 0 within 15 s. During real GVS, the current increased to the threshold value and was stable for the remaining time throughout the experimental block (15 min). In order to avoid carryover effects, we tested participants on two different days within one week.

In Experiment 2, the polarization of the electrodes was reversed: The anode was placed over the participants’ right mastoid and the cathode over their left mastoid. The mean vestibular threshold over all participants was 1.11 mA (SD =  ± 0.15). All other conditions remained unchanged.

### Procedure

**Figure 3:**
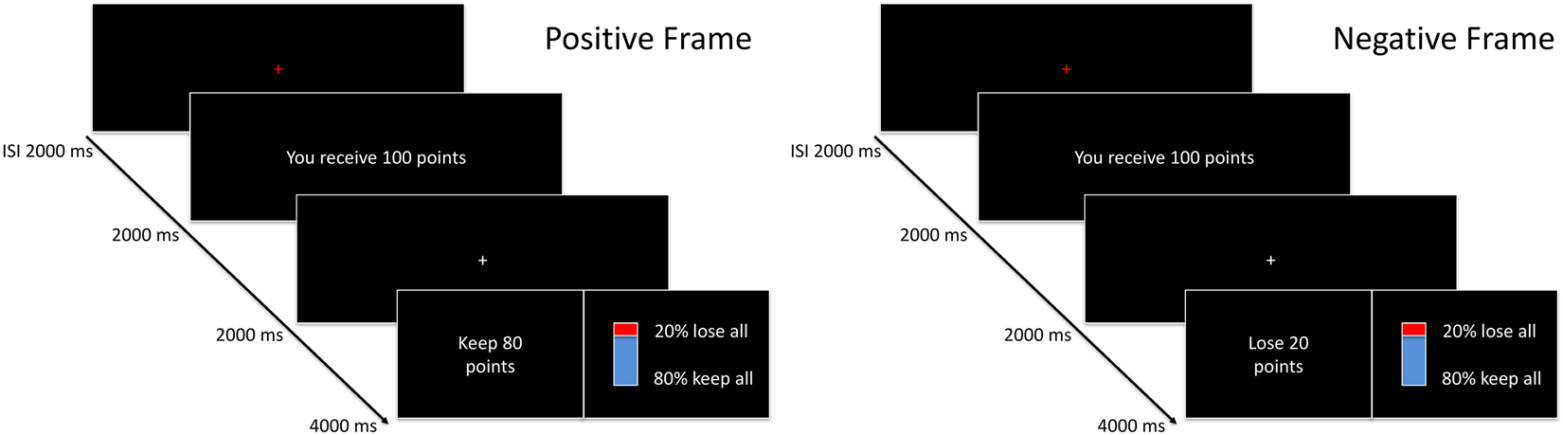
Illustration of the experimental task adapted from^29^. An initial offer was presented and the participants’ task was to decide whether they wanted to choose the safe option and keep a fixed amount of the initial points or to contend for the whole amount of the initial offer by choosing the risky option. The information in the safe option was framed either positively (“Keep 80 points” out of the initially received 100 points) or negatively (“Lose 20 points” out of the initially received 100 points).

The risky-choice framing task was adapted from DeMartino *et al*.^29^. The participants received written instructions about the task and performed a short practice session prior to the first experimental block in order to familiarize themselves with the task. In each trial, the participants were presented with an initial offer (20, 50, 80, 100 points) and then with two alternatives: They could either keep a specific amount of points (safe option) or gamble to try to keep the whole initial offer (risky option) (see Fig. 3). The safe option was either positively or negatively worded (framing condition). The participants received written instructions about the task and performed a short practice session prior to the first experimental block in order to familiarize themselves with the task. The initial offer was presented for 2000 ms, followed by a 2000-ms fixation cross after which the offer was presented again for 4000 ms. Participants were to respond within these 4000 ms. The intertrial interval was 2000 ms (see Fig. 3). The participants responded by pressing the “f” key for the safe and the “j” key for the risky option. They did not receive online feedback during the task as feedback might have influenced their decision in the next trial. Feedback was only provided at the end of each block. Each participant received five Swiss francs or one credit per hour as fixed compensation. In addition, they received additional financial compensation, the amount of which depended on their final score. Their total compensation ranged from ten francs or two credits (fixed compensation) to 20 Swiss francs (fixed compensation plus additional compensation). The probability of keeping the whole initial offer in the risky option was 20, 40, 60, or 80%. The risk level always corresponded to the safe option. For example, if participants received an initial offer of 100 points, the safe option could either be to keep 80 points for sure (positive frame) or to lose 20 points for sure (negative frame). In the risky option, they could gamble to keep the whole initial offer of 100 points (probability 80%) or to lose everything (probability 20%). Importantly, the expected outcome was identical in both conditions (positive and negative frame). Based on previous research on framing susceptibility^27^, we expected the participants to have a bias toward choosing the safe option when the safe option was positively framed and to have a bias toward choosing the risky option when the safe option was negatively framed. The participants completed a total of 64 trials in each block.

We included 32 catch trials, in which one of the two options clearly outperformed the other one (e.g., keeping 80 of 100 points for sure as compared to keeping the whole initial amount with a probability of 20%). Catch trials were included in order to control for vigilance and task commitment. If task commitment was high, the mean gambling probability was expected to be around 50%, averaged across all catch trials. Lower or higher probabilities were an indicator of low task commitment. A gambling probability of higher than 70% or lower than 30% in the catch trials were therefore set as exclusion criteria.

### Questionnaires

The participants filled out questionnaires concerning dizziness and mood after both experimental conditions. The dizziness questionnaire (Likert scale 1–10) was adapted from Lenggenhager, *et al*.^33^ and is intended to measure physiological states such as dizziness, nausea, headache, and a general feeling of discomfort during vestibular stimulation. The influence on mood states was measured using the Positive and Negative Affect Scale^37^.

### Analysis

We performed a hierarchical logistic regression with the predictors frame (positive, negative), stimulation condition (GVS, sham), and the interaction between frame and stimulation condition. The model consisted of a fixed intercept γ_00_ and a random part υ_0i_ for each participant i, fixed effects for the predictors stimulation condition γ_10_ and frame γ_20_ as well as random effects υ_1i_ and υ_2i_ for each participant i, and a fixed effect for the interaction^1^.

1 $$\mathrm{ln}({{\rm{p}}}_{({\rm{gamble}})}/{\rm{p}}{{\rm{p}}}_{({\rm{1}}-{\rm{gamble}})})={{\rm{b}}}_{0{\rm{i}}}+{{\rm{b}}}_{1{\rm{i}}}\,{\rm{Stimulation}}+{{\rm{b}}}_{2{\rm{i}}}\,{\rm{Frame}}+{{\rm{b}}}_{3{\rm{i}}}\,{\rm{Interaction}}$$

2 $${{\rm{b}}}_{0{\rm{i}}}={{\rm{\gamma }}}_{00}+{{\rm{\upsilon }}}_{0{\rm{i}}}$$

3 $${{\rm{b}}}_{1{\rm{i}}}={{\rm{\gamma }}}_{10}+{{\rm{\upsilon }}}_{1{\rm{i}}}$$

4 $${{\rm{b}}}_{2{\rm{i}}}={{\rm{\gamma }}}_{20}+{{\rm{\upsilon }}}_{2{\rm{i}}}$$

We were particularly interested in the interaction term because the interaction tests the hypothesis whether framing susceptibility was larger during GVS than during sham:

5 $${{\rm{\mu }}}_{{\rm{GVS}}\_{\rm{negative}}}-{{\rm{\mu }}}_{{{\rm{GVS}}}_{{\rm{positive}}}} > {{\rm{\mu }}}_{{\rm{sham}}\_{\rm{negative}}}-{{\rm{\mu }}}_{{\rm{sham}}\_{\rm{positive}}}$$

Using contrast coding, the hypothesis can be transformed to

6 $$1\cdot {{\rm{\mu }}}_{{\rm{GVS}}\_{\rm{negative}}}-1\cdot {{\rm{\mu }}}_{{\rm{GVS}}\_{\rm{positive}}}-1\cdot {{\rm{\mu }}}_{{\rm{sham}}\_{\rm{negative}}}+1\cdot {{\rm{\mu }}}_{{\rm{sham}}\_{\rm{positive}}} > 0$$

which describes the interaction term in the general linear model. A positive value for the interaction term would therefore indicate an increase in framing susceptibility during GVS.

The sham stimulation condition and a positive frame were set as the reference category. The data were analyzed using the software package lme4 for R^38^. Six participants in Experiment 1 and three participants in Experiment 2 failed to detect the advantageous offers in the catch trials and were therefore excluded from analysis due to low task commitment.

## Electronic supplementary material

Supplementary information
