# Supplementary material for: Framing susceptibility in a risky choice game is altered by galvanic vestibular stimulation
Source: Sci Rep. 2017 Jun 7;7:2947. doi: 10.1038/s41598-017-02909-4 (PMC5462736; doi:10.1038/s41598-017-02909-4)
Supplement: Supplementary file 1 — Supplementary information [file 41598_2017_2909_MOESM1_ESM.pdf]

Framing susceptibility in a risky choice game is altered by galvanic vestibular stimulation

Running head: GVS & framing

[1,2,3]Nora Preuss, [4,5,6]Roger Kalla, [2,4,5,6]Rene Müri, [1,2]Fred W. Mast

[1]Department of Psychology, University of Bern, 3012 Bern, Switzerland
[2]Center for Cognition Learning and Memory, University of Bern, 3012 Bern, Switzerland
[3]Department of Neuroscience, Karolinska Institutet, 17177 Stockholm, Sweden
Perception and Eye Movement Laboratory, Department of Neurology and Clinical Research, Inselspital, University Hospital Bern and University of Bern, 3010 Bern, Switzerland
[4]Division of Cognitive and Restorative Neurology, Department of Neurology, Inselspital, University Hospital Bern and University of Bern, 3010 Bern, Switzerland
[5]Department of Neurology and German Center for Vertigo and Balance Disorders, Ludwig-Maximilians-University Munich, Marchioninistr. 15, 81377 Munich, Germany
[6]Gerontechnology and Rehabilitation Group, University of Bern, 3008 Bern, Switzerland

Corresponding author:
Dr. Nora Preuss
Fabrikstrasse 8
3012 Bern
Switzerland
nora.preuss@psy.unibe.ch

Present address:
Dr. Nora Preuss
Retzius Väg 8
17177 Stockholm
Sweden

Supplementary Material

Model comparison experiment 1

Model	Predictors	Df	logLik	deviance	Chisq	df	p
0	Nullmodel	2	-1369	2737			
1	Fixed: Frame Random: Intercept	3	-1334	2667	69.94	1	6.11E-17
2	Fixed: Frame, **stimulation** Random: Intercept	4	-1334	2667	0.0001075	1	0.9917
3	Fixed: Frame, stimulation Random: Intercept, **frame, stimulation**	9	-1318	2636	30.89	5	9.86E-06
4	Fixed: Frame, stimulation, **interaction** Random: Intercept, frame, stimulation	10	-1316	2632	4.722	1	0.02978
5	Fixed: Frame, stimulation, interaction Random: Intercept, frame, stimulation, **interaction**	14	-1314	2627	4.642	4	0.326

The table shows the change in model fit depending on predictors included. Stepwise included predictors are marked in bold. Model 3 shows that the model fit significantly improved after including random slopes for frame and stimulation. Furthermore, the fit significantly improves when the interaction between frame and stimulation is included. Adding the interaction as random slope does not improve the model fit. Parameter effects are therefore reported for Model 4.

Model comparison experiment 2

Model	Predictors	Df	logLik	deviance	Chisq	df	p
0	Nullmodel	2	-1610	3220			
1	Fixed: Frame<br>Random: Intercept	3	-1575	3150	69.93	1	6.16E-17
2	Fixed: Frame, **stimulation**<br>Random: Intercept	4	-1574	3148	1.679	1	0.195
3	Fixed: Frame, stimulation<br>Random: Intercept, **frame, stimulation**	9	-1563	3127	21.22	5	0.0007364
4	Fixed: Frame, stimulation, **interaction**<br>Random: Intercept, frame, stimulation	10	-1563	3127	0.08867	1	0.7659
5	Fixed: Frame, stimulation, interaction<br>Random: Intercept, frame, stimulation, **interaction**	14	-1563	3126	0.7357	4	0.9469

The table shows the change in model fit depending on predictors included. Stepwise included predictors are marked in bold. Model 3 shows that the model fit significantly improved after including random slopes for frame and stimulation. The fit does not significantly improve neither when the interaction between frame and stimulation is included nor when adding the interaction as random slope. Parameters effects are therefore reported for Model 4 in order to make them comparable to the results of Experiment 1.
